# Autophagy inhibition improves anti-cancer drugs *via* FOXO3a activation

**DOI:** 10.18632/oncotarget.25366

**Published:** 2018-05-22

**Authors:** Brent E. Fitzwalter, Andrew Thorburn

**Affiliations:** Andrew Thorburn: Department of Pharmacology, University of Colorado School of Medicine, Aurora, CO, USA

**Keywords:** Autophagy, apoptosis, FOXO3a, PUMA, MDM2, CRISPR/Cas9

Autophagy is believed to prevent tumor initiation by promoting DNA stability, mitochondrial turnover, and tissue homeostasis. However, once a tumor has been established, autophagy allows cancer cells to overcome nutrient and environmental stresses associated with tumor cell proliferation and microenvironment [1]. Inhibiting autophagy in cancer may be a therapeutic intervention to promote cell death [2, 3], and thus understanding the molecular mechanisms linking autophagy and apoptosis is essential [4]. There are many ongoing clinical trials using autophagy inhibitors to enhance the efficacy of anti-cancer drugs, but there is limited mechanistic insight as to why they cooperate to induce more tumor cell death.

Cancer cells are, in most cases, primed to undergo apoptosis; they integrate environmental pro- and anti-apoptotic signals by using protein-protein interactions between BH3-only pro-apoptotic proteins, the apoptosis effectors BAX and BAK, and BCL-2 anti-apoptotic proteins. Cytotoxic drugs act by pushing cancer cells to commit apoptosis, and are beneficial in many cancer types and continue to be widely used [5]. Additionally, targeted therapies capitalize on specific cancer cell vulnerabilities to promote cancer cell death. It is important to understand how both cytotoxic and targeted therapies affect apoptosis threshold to commit cancer cells to undergo apoptosis.

**Figure 1:**
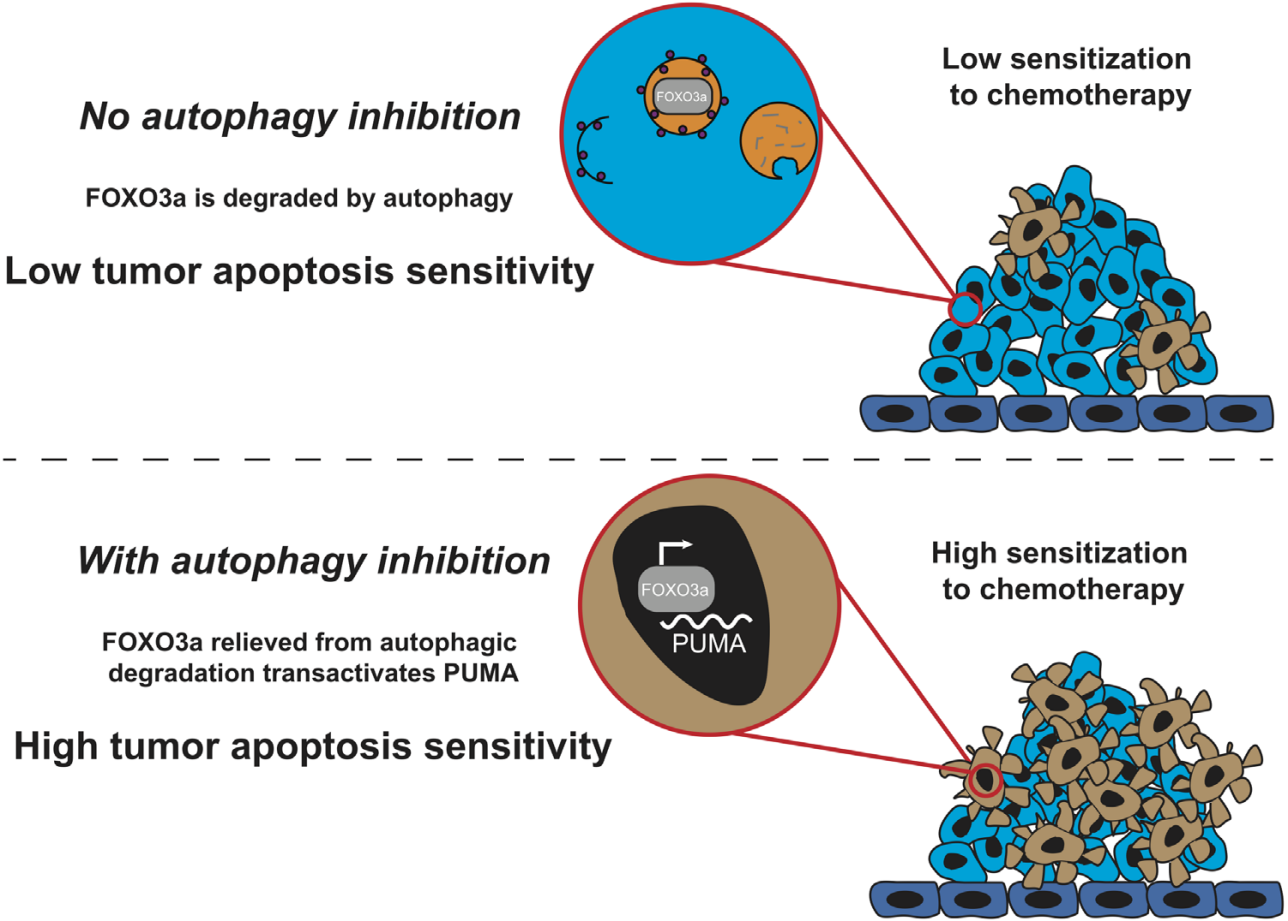
Autophagy determines sensitivity to chemotherapy Autophagic turnover of FOXO3a confers low sensitization to chemotherapy-induced apoptosis. However, upon autopagy inhibition, FOXO3a transactivation of PUMA confers high sensitization to chemotherapy-induced apoptosis.

A recent study [6] from our laboratory uncovers a transcriptional mechanism linking autophagy and apoptosis that provides a basis to inhibit autophagy to improve anti-cancer drugs (Figure 1). This study extends previous work reporting that PUMA levels increase upon autophagy inhibition, and that this increase in PUMA levels is essential for mediating apoptosis sensitization when combining autophagy inhibition with apoptosis-inducing ligands like TRAIL [7]. We found that genetic or pharmacological autophagy inhibition increases PUMA levels by increasing transactivation by the Forkhead Box transcription factor FOXO3a at a single Forkhead Response Element (FHRE) in an intron of the BBC3/PUMA gene. Importantly, CRISPR/Cas9-mediated knockout of this endogenous FHRE completely blocked the ability of autophagy inhibition to upregulate PUMA mRNA levels, but still allowed for PUMA upregulation in other contexts (p53 activation for example).

How can this mechanism be leveraged to improve cancer therapy? We showed that lysosomal disruption by chloroquine (a drug that is used in people to inhibit autophagy, but also affect other lysosomal-related processes and has lysosome independent effects as well) sensitizes cells to apoptosis upon treatment with the DNA damaging agents such as doxorubicin and etoposide, but this combinatorial effect was dependent on the ability of FOXO3a to bind to the BBC3/PUMA locus. We took the mechanism a step further to change the mode of action of an anti-cancer drug called Nutlin from cancer cell growth arrest to causing apoptosis upon autophagy inhibition. These *in vitro* data indicate that Nutlin and lysosomal inhibition cooperate to cause cancer cells to commit to apoptosis. Similar results were obtained for genetic autophagy inhibition and Nutlin combination, indicating that these effects involve macroautophagy’s ability to regulate apoptosis and not autophagy-independent effects of chloroquine. Again, these effects were dependent on the ability of FOXO3a to bind to the BBC3/PUMA locus. Consistent with these data, mice with xenograft tumors grown from parental HCT116 cells and treated with the combination of Nutlin and chloroquine had significantly better survival based on tumor burden compared to the two drugs alone. However, mice with tumors grown from cells lacking the endogenous FHRE in the BBC3/PUMA locus failed to respond to this combination treatment and displayed no better survival than parental tumors treated with Nutlin alone. These data indicate that the combination of chloroquine and Nutlin can reduce tumor burden in vivo, and that this combinatorial benefit of autophagy inhibition depends on the ability of FOXO3a to bind to the BBC3/PUMA locus in these cancer cells.

Mechanistic insight into how autophagy impacts apoptosis is just beginning to be understood. FOXO3a-mediated upregulation of BBC3/PUMA to mediate apoptosis sensitization provides a rationale for combining DNA damaging agents or Nutlin derivatives with autophagy inhibition in cancer therapy. Future studies should determine if this mechanism applies in a range of cancer types, and in the context of other anti-cancer drugs. Additionally, are different cancer sub-types dependent on other BH3-only proteins for apoptosis sensitization upon autophagy inhibition? Addressing these questions may uncover the potential of autophagy manipulation in patients to improve current therapies.
